# SARS-CoV-2, diabetes and mortality: month by month variation in mortality rate from June 2020 to June 2021

**DOI:** 10.1097/XCE.0000000000000258

**Published:** 2022-01-28

**Authors:** Adrian H. Heald, David A. Jenkins, Nadia Chaudhury, Richard Williams, Matthew Sperrin, Niels Peek, William Ollier, Kelly Bowden-Davies, Gayathri Delanerolle, Simon G. Anderson, John Martin Gibson

**Affiliations:** a The School of Medicine and Manchester Academic Health Sciences Centre, The University of Manchester, Manchester; b Department of Diabetes and Endocrinology, Salford Royal NHS Foundation Trust, Salford; c Division of Informatics, Imaging and Data Science, Faculty of Biology, Medicine and Health, University of Manchester, Manchester Academic Health Science Centre, Manchester; d NIHR Greater Manchester Patient Safety Translational Research Centre, The University of Manchester, Manchester; e University Hospitals Coventry and Warwickshire, Coventry; f Faculty of Science and Engineering, Manchester Metropolitan University, Manchester; g Department of Sport and Exercise Sciences, Musculoskeletal Science and Sports Medicine Research Centre, Manchester Metropolitan University, Manchester; h Nuffield Department of Primary Health Care Science, University of Oxford (Substantive), Oxford; i Clinical Research Facility, Southern Health NHS Foundation Trust (Honorary), Southampton, UK; j The George Alleyne Chronic Disease Research Centre, University of the West Indies, Cave Hill Campus, Bridgetown, Barbados; k Division of Cardiovascular Sciences, Faculty of Biology Medicine and Health, University of Manchester, Manchester, UK

Since early 2020, the SARS-CoV-2 virus (COVID-19) has challenged the world and perturbed our healthcare systems [1]. People with type 2 diabetes (T2D) are known to be at a higher risk of becoming unwell following COVID-19 infection when compared with people who do not have T2DM [2]. Access to longitudinal general practice electronic health records for people has allowed a greater resolution of outcomes over time for those who have contracted COVID-19.

From the start of the COVID-19 pandemic in early 2020, the monthly incidence of infection in populations has varied both between countries and within countries with a corresponding variation in mortality rate. Infection/mortality incidence has even varied at even higher resolution across counties and within cities. In this study, we have identified across a 13-month period (1 June 2020 to 30 June 2021), the relative likelihood of death each month for people previously diagnosed with T2D following confirmed COVID-19 infection. An understanding of what has happened in the past in relation to mortality from COVID-19 can inform what may happen in the future in relation to the reality of our coexistence with the COVID-19 virus.

Anonymized data on 13 807 COVID-19-infected T2D individuals were extracted from the Greater Manchester Care (UK) Record (GMCR) Graphnet database [3]. This included more than 99% of general practice patient records across the GM conurbation. Death was defined as occurring following a positive COVID-19 test up to and including 30 June 2021. Each odds ratio refers to the odds of dying once infected by COVID-19 in a given month, compared to that in the month of July 2020. The majority of deaths occurred within 28 days of confirmation of COVID-19 infection.

Using July 2020 mortality rate as the baseline (when infection rates were at their lowest in that year), for T2D individuals, the risk of dying following a recorded COVID-19 infection was comparable in January/February 2021 to that in July 2020 (Table 1), with a progressive decrease in the odds ratio of death from the start of 2021. From December 2020, the proportion of T2D individuals vaccinated against COVID-19 has steadily risen across GM for initially the first vaccination (86.18% by the end of June 2021) and then subsequently for the second vaccination (77.96% by the end of June 2021). A total of 1062 (7.7%) T2D people died during the follow-up period, following a confirmed COVID-19 infection.

**Table 1:** Mortality odds ratio each month (compared to July 2020) and cumulative proportion of type 2 diabetes individuals vaccinated from June 2020 to June 2021

Month	OR (95% CI)	% with first vaccine	% with second vaccine
Intercept	0.097 (0.059–0.149)	0	0
Jun 20	1.347 (0.77–2.422)	0	0
Aug 20	0.819 (0.435–1.551)	0	0
Sep 20	0.698 (0.411–1.225)	0	0
Oct 20	0.582 (0.365–0.976)	0	0
Nov 20	0.661 (0.413–1.11)	0	0
Dec 20	0.793 (0.486–1.352)	2.83	0.05
Jan 21	0.905 (0.57–1.513)	25.75	0.91
Feb 21	0.564 (0.333–0.987)	64.88	1.18
Mar 21	0.723 (0.399–1.33)	81.44	9.35
Apr 21	0.254 (0.083–0.64)	83.55	39.67
May 21	0.226 (0.074–0.57)	85.31	70.23
Jun 21	0.35 (0.149–0.764)	86.18	77.96

The odds ratio relates to the likelihood of death in any month compared with July 2020.

CI, confidence interval; OR, odds ratio.

The advent of treatment of unwell COVID-19-affected patients with dexamethasone has significantly reduced illness severity and mortality rates across the world [4]. While we are not in a position to ascribe causality, we propose that this may be a key determinant of the much reduced relative mortality rate in T2DM individuals after the first wave of the pandemic.

The phenomena described here are likely also related to the accumulating benefits at a population level of the UK National Health Service-led COVID-19 vaccination programme [5] from December 2020 onwards, in relation to even the first vaccine dose. Winter 2020/2021 UK lockdown will also have an impact on COVID-19 infection rates. However, we have used the ratio of mortality rate vs. confirmed COVID-19 infection rates, so the effect of lockdown measures should therefore not materially influence our findings.

Regarding limitations, our mortality data does not indicate clearly if T2D patients died of COVID-19 itself or COVID-19-related complications and specifically who, of those patients who died from December 2021 onwards, were actually vaccinated vs. those who were not. In addition, we have relied on recorded positive COVID-19 status in the GMCR. The full picture around attributable risk for patients with T2D in relation to mortality is yet to be clarified.

In conclusion, in our survey, we describe that mortality in people with T2D was no higher in early 2021 compared with mid-2020 and that mortality rates decreased through 2021. The factors underlying this likely relate to the accrual of benefits to the UK population of the COVID-19 vaccination programme and the use of dexamethasone for many hospitalized patients with a COVID-19 infection. Hopefully, further medical and therapeutic interventions will continue to improve treatments and outcomes, even as new variants of COVID-19 emerge.

## Acknowledgements

The work of D.A.J. and R.W. was funded by the National Institute for Health Research (NIHR) Greater Manchester Patient Safety Translational Research Centre. The views expressed are those of the author(s) and not necessarily those of the NIHR or the Department of Health and Social Care.

A.H.H. led on writing the original draft and contributed to the methodology and conceptualization. D.J. led on formal analysis and helped to write the manuscript at all stages. N.C. assisted with data interpretation and with writing the manuscript. R.W. accessed and verified the underlying data, supported the analysis and contributed to writing the manuscript. M.S. supervised the formal analysis and data visualization while also helping to write the manuscript at all stages. G.D. contributed to data visualization and the drawing of conclusions as did K.B.-D. S.A., N.P., W.O. and J.M.G. contributed to the conceptualization, methodology and writing the manuscript.

The data used in the analyses presented were obtained with the permission of the Greater Manchester Care Record Board and were fully anonymized before being made available to the investigators.

### Conflicts of interest

There are conflicts of interest.
